# Minichromosome maintenance proteins 2 and 5 in non-benign epithelial ovarian tumours: relationship with cell cycle regulators and prognostic implications

**DOI:** 10.1038/sj.bjc.6603992

**Published:** 2007-10-16

**Authors:** H Gakiopoulou, P Korkolopoulou, G Levidou, I Thymara, A Saetta, C Piperi, N Givalos, I Vassilopoulos, K Ventouri, A Tsenga, A Bamias, M-A Dimopoulos, E Agapitos, E Patsouris

**Affiliations:** 1 First Department of Pathology, Athens University, School of Medicine, GR-11527 Athens, Greece; 2 Department of Biological Chemistry, Athens University, School of Medicine, GR-11527 Athens, Greece; 3 Department of Therapeutics, Alexandra General Hospital, Athens University School of Medicine, GR 115 28 Athens, Greece

**Keywords:** MCM-2, MCM-5, Ki-67, ovarian low malignant potential tumours, ovarian adenocarcinomas, survival

## Abstract

Minichromosome maintenance proteins (MCM) have recently emerged as novel proliferation markers with prognostic implications in several tumour types. This is the first study investigating MCM-2 and MCM-5 immunohistochemical expression in a series of ovarian adenocarcinomas and low malignant potential (LMP) tumours aiming to determine possible associations with clinicopathological parameters, the conventional proliferation index Ki-67, cell cycle regulators (p53, p27^Kip1^, p21^WAF1^ and pRb) and patients’ outcome. Immunohistochemistry was applied in a series of 43 cases of ovarian LMP tumours and 85 cases of adenocarcinomas. Survival analysis was restricted to adenocarcinomas. The median MCM-2 and MCM-5 labelling indices (LIs) were significantly higher in adenocarcinomas compared to LMP tumours (*P*<0.0001 for both associations). In adenocarcinomas, the levels of MCM-2 and MCM-5 increased significantly with advancing tumour stage (*P*=0.0052 and *P*=0.0180, respectively), whereas both MCM-2 and MCM-5 increased significantly with increasing tumour grade (*P*=0.0002 and *P*=0.0006, respectively) and the presence of bulky residual disease (*P*<0.0001 in both relationships). A strong positive correlation was established between MCM-2 or MCM-5 expression level and Ki-67 LI (*P*<0.0001) as well as p53 protein (*P*=0.0038 and *P*=0.0500, respectively). Moreover, MCM-2 LI was inversely correlated with p27^Kip−1^ LI (*P*=0.0068). Finally, both MCM-2 and MCM-5 were associated significantly with adverse patients’ outcome in both univariate (⩾20 *vs* >20%, *P*=0.0011 and ⩾25 *vs* <25%, *P*=0.0100, respectively) and multivariate (*P*=0.0001 and 0.0090, respectively) analysis. An adequately powered independent group of 45 patients was used in order to validate our results in univariate survival analysis. In this group, MCM-2 and MCM-5 expression retained their prognostic significance (*P*<0.0001 in both relationships). In conclusion, MCM-2 and MCM-5 proteins appear to be promising as prognostic markers in patients with ovarian adenocarcinomas.

The most consistently reported significant prognostic indicators for human ovarian cancer are disease stage, tumour grade, histologic type and extent of surgical cytoreduction (Ozols, 2002). However, there remains a significant degree of biologic heterogeneity even within the same prognostic subgroups, hampering the accurate prediction of clinical behaviour in individual cases (Ozols, 2002). Thus, gaining insight into the molecular determinants of the biologic behaviour of ovarian cancer is imperative to identify groups of patients with a particularly poor prognosis.

The rapidly advancing understanding of mammalian DNA replication has given rise to a new generation of proliferation markers. Recently, positive and negative regulators of DNA replication have emerged as novel tumour biomarkers that may potentially be used for screening, estimation of prognosis and assessment of treatment response for a range of tumour types (Tachibana *et al*, 2005). Minichromosome maintenance (MCM) proteins drive the formation of prereplicative complexes (PRCs), which is the first key event during G_1_ phase (Tye, 1999). In all eukaryotic cells, initiation of DNA synthesis is a complex multistep process tightly coupled to progression through the cell cycle (Ritzi and Knippers, 2000). In the first step, during the G_1_ phase key replication initiation factors, including the origin recognition complex, the Cdc6 protein and the MCM proteins 2–7 are recruited into PRCs at future replication origins, establishing the competence of particular chromatin regions for initiation of DNA synthesis (Tye, 1999; Labib and Diffley, 2001; Lei and Tye, 2001; Yu *et al*, 2004b). The MCMs are highly conserved proteins presumed to act as an enzymatically active helicase (Labib and Diffley, 2001). In the second step, unwinding of replication origins and establishment of bi-directional replication forks take place to control entry into the S phase (Labib and Diffley, 2001). As the DNA is replicated, MCM proteins gradually dissociate from chromatin, ensuring that each region of DNA is replicated only once during a single cell cycle, because replicated DNA lacks functional PRCs (Labib and Diffley, 2001). As the cells leave the cell cycle to enter into the quiescent, differentiated and senescent states, the Cdc6 and MCM components of the replication initiation pathway are downregulated (Stoeber *et al*, 2001).

Recently, several groups have reported that MCM immunoreactivity is a specific and accurate marker for proliferating cells (Todorov *et al*, 1998; Freeman *et al*, 1999; Heidebrecht *et al*, 2001; Kodani *et al*, 2001; Stoeber *et al*, 2001). Other advantages of MCMs are that they do not detect cells undergoing DNA repair and they are not downregulated in proliferating cells by nutritional deprivation, which may be operating regionally in solid tumours (Osaki *et al*, 2002). Also, their function has been well characterised in several *in vitro* systems (Tye, 1999) representing a point of convergence for numerous signalling pathways involved in cell growth (Stoeber *et al*, 2001) and their levels change little during the cell cycle, decreasing markedly in cells with a lower proliferation rate (Todorov *et al*, 1994).

To the best of our knowledge, there is only one previous study assessing MCMs in ovarian neoplasia (Scott *et al*, 2004). However, this study has focused solely on serous neoplasms and has investigated only MCM-2 protein expression. More importantly, nothing is known about the prognostic utility of MCM proteins in ovarian cancer. The aim of the present study was to investigate the immunohistochemical expression of MCM-2 and MCM-5 proteins in a series of low malignant potential (LMP) tumours and ovarian carcinomas of all histologic types and to determine for the first time the possible associations with clinicopathologic parameters, the conventional proliferation index Ki-67, other cell cycle regulators (p53, p27^Kip1^, p21^WAF1^ and pRb) and patients’ outcome.

## MATERIALS AND METHODS

### Study populations

#### Patient population

This is a retrospective study of 128 consecutive cases of epithelial ovarian tumours of LMP and adenocarcinomas, diagnosed and treated at Alexandra General Hospital and Iasso Gynaecologic Institute of Athens between 1989 and 1999, for whom paraffin-embedded tissue and clinical information were available. Eighty-five specimens were defined as ovarian adenocarcinomas, whereas 43 specimens fulfilled the criteria of LMP tumours (Scully and Sobin, 1987; Russel, 1994). Histologic classification of tumours was carried out according to the World Health Organization System (Scully and Sobin, 1987). Poorly differentiated adenocarcinoma was the diagnosis for those carcinomas that did not show evident cellular differentiation. Adenocarcinomas were graded as well, moderately and poorly differentiated (Scully and Sobin, 1987) and patients were assigned a clinical stage according to the International Federation of Gynecology and Obstetrics (Cancer Committee of FIGO, 1986) standards. Surgical and pathologic findings and postoperative abdomino-pelvic computerised tomography (CT) scans were used to determine the FIGO stage for the ovarian adenocarcinomas and the residual disease after the initial surgery. Persistence of tumour masses of <2 cm was defined as minimal residual disease, whereas the presence of masses of >2 cm in diameter was defined as bulk residual disease (Anttila *et al*, 1999; Sengupta *et al*, 2000).

All patients with carcinomas had undergone total abdominal hysterectomy with bilateral salpingo-oophorectomy followed by chemotherapy. None had received preoperative chemotherapy or radiotherapy. The clinicopathologic characteristics of these patients are summarised in Table 1. Follow-up information was available in 79 patients. By the time this study was undertaken, 26 patients had died of their disease after a median survival of 15.6 (range 1–60) months. The median follow-up for the remaining 53 patients was 43 (range 11.7–126) months.

### Validation group

An independent set of patients with ovarian adenocarcinoma was used to validate our results of univariate survival analysis and test the validity of the chosen cutoff values for the expression of MCM-2 and MCM-5 proteins. The results of univariate survival analysis for MCM-2 and MCM-5 expression in the population group were used to calculate the required number of patients in the validation group for an adequately powered analysis (90%) (Lachin and Foulkes, 1986). Lachin (1981) have showed that the equation relating total sample size and power for a univariate survival analysis test in simple sets is given by the following equation: 
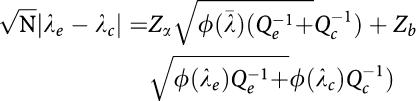
 where *λ*e, *λ*_c_ the respective hazard ratios in the two groups, *Q*_*i*_=*n*_*i*_/*N*, *λ¯=Q*_*c*_*λ*_*c*_*+Q*_*e*_*λ*_*e*_ and Za, Zb the standard normal derivatives at levels a and b, respectively.

Given that our study implies non-uniform patients’ entry and consequently follow-up, this type can be transformed when we suppose the following exponential entry distribution (Lachin and Foulkes, 1986). 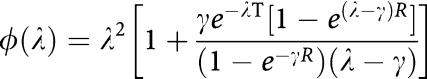


Sample size and power can be calculated by substituting this type into the above.

The validation group consisted of 45 patients with primary ovarian adenocarcinoma, diagnosed and treated at Alexandra General Hospital between 1994 and 2004, for whom paraffin-embedded tissue was available. Follow-up period ranged from 1.3 to 124 months (median: 23.96 months). During this period, 16 disease-specific deaths were recorded, whereas the median (range) follow-up for the remaining 29 cases was 28.5 (9–124) months. The clinicopathologic data of the validation group are summarised in Table 2.

### Processing of specimens and immunohistochemistry

Tissues were fixed immediately after removal in 10% buffered formalin and processed to paraffin wax. Four micrometres serial sections were cut from each specimen on Superfrost plus glass slides and left to dry overnight at 37°C. In addition, from our previous investigations (Korkolopoulou *et al*, 2002; Konstantinidou *et al*, 2003; Vassilopoulos *et al*, 2003), results regarding the expression of p53, p27^Kip1^, pRb, p21^WAF1^ and Ki-67 were available in all cases. The following monoclonal antibodies were used: (1) MCA 1859 for MCM-2 (clone CRCT2.1/D1.9H5) (Serotec Ltd, Oxford, UK, diluted 1 : 500 overnight) and (2) MCA 1860 for MCM-5 (clone CRCT5.1/A2.7A3) (Serotec Ltd, Oxford, UK, diluted 1 : 200 overnight). For both antibodies, antigen retrieval with microwaving for 30 min, at 750 W, in citrate buffer pH 6.0 was required. The peroxidase-polymer-based method was used with DAKO Envision kit (DAKO, Carpinteria, CA, USA). Known positive controls (i.e. a normal tonsil) as well as negative controls (sections in which anti-MCM-2 and anti-MCM-5 antibodies were substituted by non-immune mouse serum) were also stained in each run.

Staining for MCM-2 and MCM-5 was assessed blindly (i.e. without any knowledge of the clinical data) by two observers. A difference of greater than 5% between the two assessments was observed in 7.8% (10/128) and 10.15% (13/128) of cases, respectively. In these cases, slides were reviewed jointly and a consensus was reached. Nuclei from about 1000 tumour cells from systematically randomised fields (× 40) throughout the entire section were counted and the labelling index (LI) was calculated as the percentage of labelled nuclei out of the total number of tumour cells counted. Whenever heterogeneous staining was encountered, counting was performed in areas of highest density of labelled cells, identified at medium magnification (× 20), because it has been proposed that these tumour areas are most likely to be of biologic significance (Wharton *et al*, 2001; Rodins *et al*, 2002). All clearly identifiable nuclear staining beyond background was recorded as positive for MCM-2 and MCM-5. No endothelial or lymphoid cells were included in the counts even though they expressed MCM-2 and MCM-5.

### Cell culture

MDAH-2774 cells were serially passaged as monolayer cultures in RPMI medium, supplemented with 10% FCS, 1% L-glutamine, 50 mg ml^−1^ streptomycin and 50 U ml^−1^ penicillin (Gibco-BRL/Life Technologies, Eggenstein, Germany). The cell-culture flasks (Nunc, Wiesbaden, Germany) were incubated in a humidified atmosphere containing 5% carbon dioxide at 37°C. Cells grown for the appropriate time interval were washed with phosphate-buffered saline (PBS, pH 7.4; 140 mM NaCl; 6.5 mM Na2HPO4; 2.5 mM KCl; 1.5 mM KH2PO4) and harvested by a 3-min treatment with 0.25% trypsin/0.02% EDTA (Gibco-BRL/Life Technologies) in PBS. Equal amounts of culture medium were added and cells were centrifuged for 5 min at 600 **g** and resuspended in PBS for further processing.

#### Cell synchronisation by nocodazole treatment

Mitotic arrest was induced by adding 50 ng ml^−1^ nocodazole (Sigma, Deisen-hofen, Germany) from a 500 mg ml^−1^ stock solution in dimethyl sulphoxide (DMSO) to the culture medium and incubating the cells for 24 h, as described previously (Endl and Gerdes, 2000). After 24 h treatment, metaphase cells in G_1_ were immediately harvested by gentle shaking and washed two times with PBS.

#### Immunofluorescence and immunohistochemistry

As soon as synchronised and unsynchronised cells have been harvested, they were trypsinised, plated (at 1 × 10^5^ cells ml^−1^) on glass slides in six-well cell culture plates and fixed by gently removing the culture medium and adding 2% formaldehyde in PBS (pH adjusted to 7.4) for 10 min on ice. Cells were permeabilised by incubation in 0.25% Triton X-100 for 5 min on ice and washed twice with PBS containing 4% BSA.

Immediately after this procedure has been completed slides for immunofluorescence were incubated overnight with Ki-67, MCM-2, or MCM-5 antibodies at 4°C, diluted 1 : 20 in a humidified chamber, followed by washing in PBS supplemented with 4% BSA and incubation with FITC-conjugated anti-mouse secondary antibody diluted 1 : 200 (Santa Cruz Biotechnology, Santa Cruz, CA, USA) for 1 h at 4°C. For immunohistochemistry, the same procedure was followed, substituting FITC-conjugated secondary antibody with DAKO Envision kit, as used for the immunohistochemistry of tissue specimens. Ki-67, MCM-2 and MCM-5 dilutions in this instance were 1 : 300, 1 : 400 and 1 : 100, respectively. For both immunohistochemistry and immunofluorescence, each experiment was repeated three times. After washing with PBS, all slides were finally incubated in glycerol and examined under the microscope. Immunofluorescence slides were photographed on fluorescent microscope. At least 300 cells were examined under the microscope at medium and high-power magnification and the percentage of cells displaying nuclear immunoreactivity or immunofluorescence for MCMs and Ki-67 was estimated. Images were stored as TIF files.

### Statistical analysis

The normality of distributions was tested with the Kolmogorov–Smirnov test. Associations between MCM-2 or MCM-5 LI and histologic type, grade, stage and residual disease were assessed with Mann–Whitney *U*-test and Kruskal–Wallis analysis of variance as appropriate. The correlation between MCM-5 protein and histologic stage when poorly differentiated and unspecified tumours were excluded from analysis was tested using Kruskal–Wallis ANOVA using the Bonferroni correction for multiple comparisons. The interrelations among MCM-2, MCM-5, Ki-67, p53, p21^WAF1^, p27^Kip1^ and Rb, in LMP tumours and adenocarcinomas, were analysed with Spearman's rank correlation coefficient. The Cuzick extension of the Wilcoxon's rank sum test was used to examine the trend of MCM-2 and MCM-5 LI in relation with increasing tumour stage. The difference between MCM-2 or MCM-5 LI and Ki-67 LI in ovarian adenocarcinomas was examined with Wilcoxon's signed rank paired test and in MDAH-2774 with the null hypothesis.

Survival analysis was restricted to adenocarcinomas, given that LMP tumours follow a substantially more favourable clinical course. The prognostic effect of various parameters (i.e. age, histologic type, grade, FIGO stage, volume of residual disease, type of chemotherapy, LI, MCM-2 LI and MCM-5 LI on clinical outcome, i.e. death of disease) was tested by plotting survival curves according to Kaplan–Meier method and comparing groups using the log-rank test, as well as by multivariate analysis. Patients dying of other causes during the follow-up period were treated as censored data. In univariate analysis, the numerical variables age, MCM-2 LI and MCM-5 LI, were categorised on the basis of the median value. In multivariate analysis, we also included Ki-67, p21^WAF1^, p27^Kip1^, p53 and pRb expression to examine whether their presence may influence the prognostic impact of MCM proteins. To avoid any ‘data-driven’ categorisation, numerical variables were entered in multivariate analysis as continuous variables. Given that MCM-2 and MCM-5 were strongly interrelated, their significance as independent prognosticators was tested in separate models.

Statistical analysis was performed using the SPSS for Windows Software (SPSS Inc., Chicago IL, USA). A *P*-value of less than or equal to 0.05 was considered indicative of a statistically significant difference.

## RESULTS

### MCM-2 and MCM-5 expression in LMP tumours and ovarian adenocarcinomas

**Figure 1:**
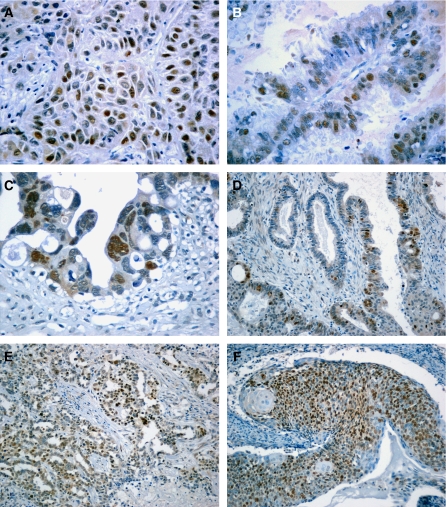
Immunohistochemical expression of MCM-2 and MCM-5 in ovarian adenocarcinomas. (**A**) MCM-2 protein expression in many nuclei of a grade 3 endometrioid ovarian adenocarcinoma (original magnification × 400). (**B**) Minichromosome maintenance protein-2 protein expression in a grade 1 ovarian adenocarcinoma: scattered nuclei stained positive (original magnification × 400). (**C**) Minichromosome maintenance protein-5 protein expression in a grade 3 serous ovarian adenocarcinoma (original magnification × 400). (**D**) Moderate MCM-5 immunopositivity in a grade 2 endometrioid ovarian adenocarcinoma (original magnification × 200). (**E**) Extensive MCM-2 positivity in a grade 2 serous ovarian adenocarcinoma (original magnification × 200). (**F**) Diffuse MCM-5 positivity in a grade 3 endometrioid ovarian adenocarcinoma (original magnification × 200).

Immunoreactivity for MCM-2 and MCM-5 was seen in all cases ranging from 1 to 82% (median: 15%) and from 1 to 75% (median: 20%), respectively (Figure 1). The pattern of staining was mostly nuclear, although a faint cytoplasmic staining was seen in some cases, which was disregarded during evaluation, conforming with previously published data (Meng *et al*, 2001). Minichromosome maintenance protein-2- and MCM-5-positive cells were randomly distributed throughout the neoplastic population in 110 and 112 cases, respectively. A statistically significant positive correlation was established between MCM-2 and MCM-5 protein levels (*P*<0.0001).

The median MCM-2 and MCM-5 LIs were significantly higher in adenocarcinomas (20 and 24%, respectively) compared to LMP tumours (5 and 6%, respectively) (Mann–Whitney *U*-test, *P*<0.0001 for both associations). When comparisons between LMP cases and carcinomas were made for serous and mucinous types separately, statistically significant differences were recorded for both MCM-2 (Mann–Whitney *U*-test, *P*<0.0001 for serous and *P*=0.0070 for mucinous tumours) and MCM-5 protein expression (Mann–Whitney *U*-test, *P*<0.0001 for serous and *P*=0.0020 for mucinous tumours; Table 2). Within the adenocarcinomas only, MCM-5 (but not MCM-2) protein expression was also related to the histologic type (Kruskal–Wallis ANOVA, *P*=0.0349), the highest levels of immunoreactivity being recorded in poorly differentiated and nonspecified categories (Table 3). However, when poorly differentiated and unspecified cases, which are regarded as high-grade disease, were removed from analysis, the relationship failed to reach statistical significance (Kruskal–Wallis ANOVA, Bonferroni correction, *P*=0.0856).

### Minichromosome maintenance protein-2 and MCM-5 expression in ovarian adenocarcinomas in relation to clinicopathologic parameters

The rate of MCM-2 or MCM-5 positivity significantly increased with increasing tumour grade (1 *vs* 2 *vs* 3, Kruskal–Wallis test: *P*=0.0002 and 0.0006, respectively; Table 4) and the presence of residual disease (Mann–Whitney *U*-test: *P*=0.0010 in both relationships). A statistically significant positive association emerged between MCM-2 and MCM-5 LI and advancing tumour stage (I&II *vs* III *vs* IV, Kruskal–Wallis test: *P*=0.0062 and 0.0018, respectively; Table 4). The Cuzick extension of the Wilcoxon's rank sum test confirmed the presence of a significant trend of increasing MCM-2 and MCM-5 expression with increasing tumour stage (*P*=0.0100 and 0.0300, respectively).

### Relationship of MCM-2 LI and MCM-5 LI with proliferation rate

**Figure 2:**
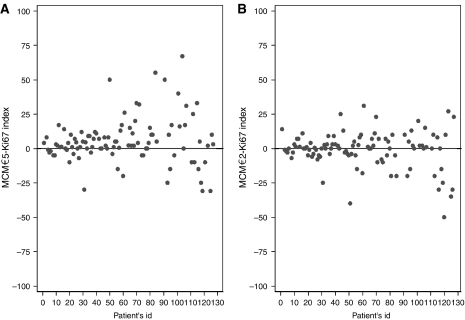
Scatter plot indicating the difference (**A**) between MCM-5LI and Ki-67LI and (**B**) between MCM-2LI and Ki-67LI. It is obvious that in the majority of cases the difference between MCM-5 and Ki-67 levels is >0.

A strong positive correlation was established between MCM-2 or MCM-5 expression level and Ki-67 LI (Spearman's rho=0.7214, *P*<0.0001 and rho=0.6449, *P*<0.0001, respectively), which remained after stratification of our cases into borderline (*P*<0.0001 and 0.0038, respectively) or carcinoma categories (*P*=0.0242 and <0.0001, respectively). Within both the LMP and adenocarcinoma groups MCM-5 (but not MCM-2)-positive cells tended to outnumber Ki-67-positive cells (Wilcoxon's signed rank paired test *P*=0.0300 and 0.0206, respectively, Figure 2). Within the adenocarcinomas statistically significant differences between MCM-5 and Ki-67 levels were recorded only in grade I tumours (*P*=0.0049).

### Minichromosome maintenance protein-2, MCM-5 and Ki-67 expression in MDAH-2774 cells

**Figure 3:**
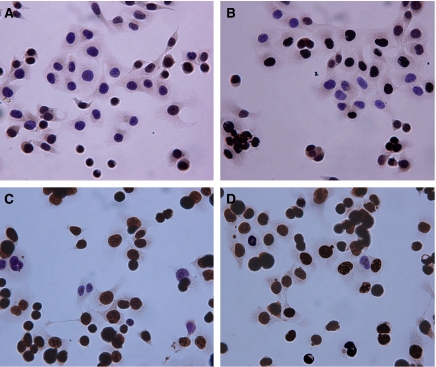
Immunohistochemical expression of MCM-5 and Ki-67 in an ovarian carcinoma cell line: (**A**) Ki-67 expression in asynchronous cell culture; (**B**) MCM-5 expression in asynchronous cell culture; (**C**) Ki-67 expression in G_1_ synchronised cell culture; (**D**) MCM-5 expression in G_1_ synchronised cell culture (× 400).

In asynchronous cell culture, a greater number of cells expressed MCM-2 and MCM-5 than Ki-67, the corresponding LIs being 95, 98 and 88%, respectively (MCM-2 *vs* Ki-67 *P*=0.0759, MCM-5 *vs* Ki-67 *P*=0.0056). This difference remained and even became greater in G_1_ arrested synchronised cells (MCM-2 *vs* Ki67 *P*<0.0001, MCM-5 *vs* Ki-67 *P*=0.0039). The LIs in this instance were 79, 70 and 50%, respectively, significantly lower than those in asychronous cell culture (MCM-2 *P*=0/0008, MCM-5 *P*<0.0001, Ki-67 *P*<0.0001, Figure 3).

### Relationship of MCM-2 and MCM-5 expression with p27^Kip1^, p21^WAF1^, p53 and pRb

In LMP tumours, the levels of MCM proteins did not correlate with either p27^Kip1^, pRb, p21^WAF1^ or p53 expression (*P*>0.10). Also, in ovarian carcinomas, no association was established between p21^WAF1^ and MCM-2 or MCM-5 LIs (*P*>0.10). However, higher MCM-2 or MCM-5 LIs were observed in ovarian adenocarcinomas with p53 Lis >10% (Kruskal–Wallis ANOVA *P*=0.0022 and 0.0300, respectively).

**Figure 4:**
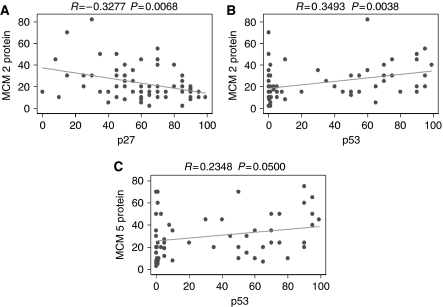
Scatter plot indicating the correlation between MCM-2 LI and p27 LI (**A**) MCM-2 LI and p53 LI (**B**) and MCM-5 LI and P53 LI (**C**).

In carcinomas, a statistically significant inverse relation was observed between MCM-2 and p27^Kip1^ levels (Spearman's rho=−0.3277, *P*=0.0068, Figure 4A). Moreover, statistically significant positive associations emerged between MCM-2 or MCM-5 LI and p53 expression (Spearman's rho=0.3493, *P*=0.0038, Figure 4B and rho=0.2348, *P*=0.0500, Figure 4C, respectively).

### Survival analysis: population group

**Figure 5:**
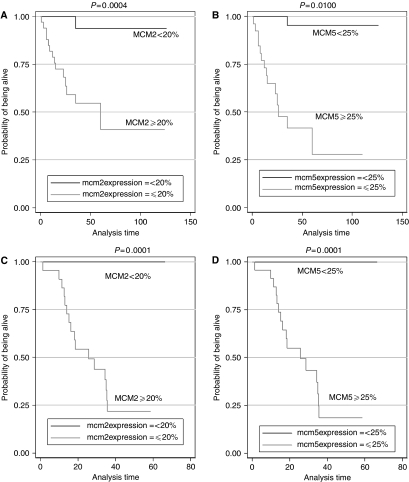
Kaplan–Meier curves of overall survival according to MCM-5 and MCM-2 protein expression in both the population group (**A**, **B**) and the validation group (**C**, **D**).

In univariate analysis, the parameters adversely affecting survival were high grade (*P*=0.0330), advanced stage (*P*=0.0193), the presence of bulk residual disease (*P*=0.0001), increased (⩾20%) MCM-2 expression (*P*=0.0004) and increased (⩾25%) MCM-5 expression (*P*=0.0100) (Figure 5A and B), whereas histologic type attained a marginal significance in this regard (*P*=0.0900).

In multivariate Cox's proportional hazard analysis of overall survival including MCM-2 LI, stage, grade, histology, chemotherapy and the presence of residual disease, MCM-2 protein expression emerged as an independent predictor of adverse outcome (*P*=0.001) along with advanced stage of the disease (Table 5, model A1). When multivariate analysis was repeated for MCM-5 and the aforementioned clinicopathological parameters, MCM-5 LI emerged as the only independent predictor of survival (*P*=0.0360; Table 5, model B1) When the other cell cycle regulators were also introduced in the models, MCM-2 remained an independent predictor of survival (*P*<0.0001) along with the stage of the disease and p21^WAF1^ expression (Table 5, model A2), whereas MCM-5 remained the only independent predictor of survival against all other parameters examined (*P*=0.0220; Table 5, model B2).

### Univariate survival analysis: validation group

To detect a difference of 0.5282 between 0.9357 and 0.4093 – the probability of surviving in high MCM-2 (⩾20%) and low MCM-2 (<20%) expression group after 60.00 months, as calculated in the population group – using a two-sided log-rank test and to achieve 90% power at a 0.05 significance level, 42 patients would be needed. Accordingly, for the detection of a difference of 0.6745 between 0.9524 and 0.2779 – the probability of surviving in high MCM-5 (⩾25%) and low MCM-5 (<25%) expression group after 60.00 months, as calculated in the population group – using a two-sided log-rank test and to achieve 90% power at a 0.05 significance level 29 patients would be needed. Consequently, we formed a random independent adequately powered validation group of 45 patients with ovarian carcinoma.

Using the optimal cutoff of 20% for MCM-2 expression and 25% for MCM-5 expression the 45 patients in the validation group were stratified into low and high expression group. The overall survival was significantly lower in the high MCM-2 expressor group when compared to that in the low MCM-2 expressor group (log-rank test, *P*<0.0001). The same applied to patients with high MCM-5 expression ((⩾25%) compared to those with low MCM-5 expression (log-rank test, *P*<0.0001)) (Figure 5C and D).

## DISCUSSION

In the present study, we have clearly demonstrated the nuclear localisation of MCM-2 and MCM-5 proteins in ovarian neoplasms. A faint cytoplasmic immunostaining was also seen in some cases, but was not taken into accounting statistical analysis, in keeping with previous observations in urothelial and prostatic carcinomas (Meng *et al*, 2001; Korkolopoulou *et al*, 2005). Studies in the budding yeast have documented the distribution of MCM-2 and MCM-3 proteins in both the cytoplasm and nucleus in relatively constant levels throughout the cell cycle (Tye, 1999). However, about 5–10% of these proteins were tightly associated with chromatin from early G_1_ phase to the beginning of S phase, when replication initiation occurs (Blow and Laskey, 1988), suggesting that active MCM complex is likely to be localised to chromatin. Yet, nuclear expression and activity are not synonymous terms as nuclear MCM is entirely unbound in G_2_ (Tye, 1999).

A statistically significant positive correlation was established between MCM-2 and MCM-5 protein levels suggesting the requirement of both proteins to create a functional complex. In this context, various methods have documented the physical interactions among members of the MCM family in man and other organisms (Richter and Knippers, 1997). Thus, immunoprecipitation of one of the MCM proteins often leads to the co-precipitation of all six members of the MCM family (Kubota *et al*, 1997; Thömmes *et al*, 1997). Chromatin immunoprecipitation in HeLa cells at the G_1_/S-phase transition indicates that all six MCMs colocalise on shared DNA fragments of 500 base pairs, advocating that these proteins are bound to chromatin as a multimeric complex containing all six subunits (Ritzi *et al*, 1998).

Given that MCMs play a critical role in initiation of DNA synthesis and DNA replication must precede each cell division, their expression is expected to correlate with cell proliferation. Along this line, accumulating evidence highlights the value of MCM protein expression as a novel indicator of cell proliferation (Facoetti *et al*, 2006). Indeed, in our study, strong correlations emerged between MCM-2 or MCM-5 LIs and the conventional proliferation index Ki-67, which remained even after stratification of our cases into borderline or carcinoma categories. Our work in an ovarian cancer cell line stained immunohistochemically and by immunofluorescence for MCM-2, MCM-5 and Ki-67 verified that MCM-positive cells (especially MCM-5) outnumbered Ki-67-positive cells in unsynchronised cells (98 *vs* 88%) and that this difference became even greater in synchronised cells arrested in G_1_ (79 *vs* 55%). However, and in keeping with our previous observations in muscle-invasive urothelial carcinomas (Korkolopoulou *et al*, 2005), median values of MCM-5 were significantly higher than those of Ki-67 in ovarian carcinomas as well as in LMP tumours. It has been claimed that MCMs represent a potentially more accurate means of determining the proliferative fraction within a tumour than the conventional proliferation indices probably because the latter fail to label cells in early G_1_ (Osaki *et al*, 2002; Korkolopoulou *et al*, 2005; Schrader *et al*, 2005). However, the discrepancy between MCMLIs and Ki-67 LI might be a reflection of the fact that Ki-67 is downregulated early in the differentiation programme, whereas MCMs are downregulated later, when the cells adopt a terminally differentiated phenotype (Eward *et al*, 2004; Kingsbury *et al*, 2005). Thus, the ‘improved’ sensitivity of MCMs as markers of proliferating cells could reside in the identification of differentiating cells, which is consistent with our finding that the highest difference among MCM-5 and Ki-67 is recorded within the grade I (i.e. the well-differentiated) tumours. Therefore, the higher growth fraction identified by MCMs appears to arise either from the fact that MCMs identify all cell cycle phases, as opposed to the low expression of Ki-67 in early G_1_ and M phase, or from the different kinetics of MCMs and Ki-67 downregulation during exit form the mitotic cycle into the differentiated state.

As mentioned above, during early G_1_, the MCM loading factors Cdc6 and Cdt1 recruit the MCM proteins to chromatin near origins of replication where they remain assembled until late G_1_ phase. S phase is then triggered by high cyclin A/CdK2 and Dbf4/Cdc7 levels that activate the complex of MCM proteins and chromatin (Tachibana *et al*., 2005). On the other hand, in most normal tissues, p27^Kip1^ negatively regulates cell proliferation by inhibiting CdKs (Schmider-Ross *et al*, 2006). Thus, the demonstration in this study of an inverse correlation between p27^Kip1^ and MCM-2 is in alignment with the underlying mechanism of regulation of cell proliferation.

Although p21^WAF1^ also constitutes a CdK inhibitor, no association was established between p21^WAF1^ and MCM-2 or MCM-5 LIs. However, the inhibitory function of p21^WAF1^ on DNA synthesis requires the formation of quarternary complexes composed of cyclins, CdKs and PCNA and is thought to be stoichiometrically regulated being exerted only when p21^WAF1^ is in molar excess (reviewed by Vassilopoulos *et al*, 2003). If the ratio of p21^WAF1^ to CdK is less than one, p21^WAF1^ serves only as an assembling factor for CdK complex and does not inhibit CdK activity.

In LMP tumours, the levels of MCM proteins did not correlate with either p27^Kip1^ or p21^WAF1^ in keeping with our previous observations concerning Ki-67, p21^WAF1^ and p27^Kip1^ expression in LMP ovarian tumours (Korkolopoulou *et al*, 2002, Vassilopoulos *et al*, 2003). Palazzo *et al* (2000) noted a similar lack of correlation between proliferation rate and the levels of p21^WAF1^ in LMP tumours. The authors claimed that this finding was to be expected in tumours with a low proliferation index as well as in normal tissues and benign tumours. The low Ki-67 levels in LMP tumours might indicate that most cells are still able to exit from the cell cycle into G_0_, as opposed to malignant tumours. Following this line of argument, this group of authors (Palazzo *et al*, 2000) attributed the lack of p21^WAF1^ in non-cycling cells to degradation of the protein, once the cell has entered a cell cycle exit state.

Given that p53 immunoreactivity in most carcinomas has been regarded as a surrogate marker for, although not a proof of, gene mutation or inactivation, the observed correlations between MCM-2 or MCM-5 on one hand and p53 on the other hand might be indicative of the positive effect exerted by mutant-type p53 on cell cycle progression. This concept is reinforced by the fact that higher MCM-2 and MCM-5 LIs were observed in the 32 (47.8% of cases) ovarian adenocarcinomas with p53 LI more than 10%, a cutoff that that has been shown to be efficient for the identification of p53 mutations in paraffin blocks staining (Banks *et al*, 1986). According to a recent study, p53 mutation prevalence estimates were 45, 5 and 1%, respectively, for invasive, LMP and benign ovarian tumours (Reles *et al*, 2001; Kmet *et al*, 2003). As opposed to carcinomas, no relation was established between p53 and cell proliferation markers in LMP tumours.

Minichromosome maintenance protein-2 and MCM-5 proteins demonstrated increased expression in ovarian adenocarcinomas as opposed to LMP tumours. These differences remained significant when serous and mucinous neoplasms were investigated separately. Moreover, no difference existed in MCMs expression between serous and mucinous LMP tumours, whereas in adenocarcinomas, a higher level of MCM-5 expression was observed in poorly differentiated category as compared with the remaining histologic types. However, the fact that this correlation did not retain its statistical significance when the poorly differentiated category was removed from analysis could possibly reflect the significant correlation between histologic grade and MCM-5 protein, as poorly differentiated tumours are regarded as high-grade disease. Our findings support and expand the previously reported increase of MCM-2 expression during the progression from normal ovary through serous cystadenoma and serous borderline tumours to serous cystadenocarcinomas (Scott *et al*, 2004). Consistent with these findings, MCM genes have also been found to be upregulated at the mRNA level in a range of malignancies by expression microarray analysis (Rosenwald *et al*, 2003; Neben *et al*, 2004; Yu *et al*, 2004b). No particular MCM protein appears to be upregulated in isolation, which is consistent with their function as a heterohexameric complex (Tachibana *et al*, 2005).

In ovarian adenocarcinomas, MCM-2 and MCM-5 expression increased with increasing tumour grade, advancing stage and the presence of bulk residual disease. Significant associations between MCM overexpression and high grade have also been described in prostate (Meng *et al*, 2001), urothelial (Korkolopoulou *et al*, 2005) and renal carcinomas (Rodins *et al*, 2002) as well as in oligodendrogliomas (Wharton *et al*, 2001). Moreover, *in vitro* studies have clearly shown a dramatic decrease in the levels of MCM-2 mRNA as well as of MCM-3 protein during the differentiation of human myeloblast HL60 cells (Philipova *et al*, 1991; Musahl *et al*, 1998), respectively, which support our findings. Relevant to this issue is the observation that overexpression of p21^WAF1^ or p27^Kip1^ in the promyelocytic leukemia cell line accelerates its lineage-specific differentiation (Wang *et al*, 1998; Zhou *et al*, 1999).

A main scope of our study was to investigate for the first time the prognostic relevance of MCM-2 and MCM-5 expression in ovarian adenocarcinomas. In both univariate and multivariate survival analysis, overexpression of each protein associated significantly with poor overall patients’ survival. More importantly, the results of univariate survival analysis were validated in an independent set of patients, using the same cutoff points of MCM expression as in the population study. The confirmation of the adverse prognostic effects of a notorious group of universally established prognostic factors (grade, stage, residual disease) proves that our cohort was quite representative and that survival analysis was valid.

Immunohistochemical studies and expression microarray analyses have independently identified MCM proteins as powerful indicators of worse clinical outcome in various tumour types (Meng *et al*, 2001; Ramnath *et al*, 2001; Wharton *et al*, 2001; Hunt *et al*, 2002; Rodins *et al*, 2002; van ‘t Veer *et al*, 2002; Gonzalez *et al*, 2003; Kato *et al*, 2003; Kodani *et al*, 2003; Kruger *et al*, 2003; Rosenwald *et al*, 2003; Sotiriou *et al*, 2003; Hashimoto *et al*, 2004; Neben *et al*, 2004; Yu *et al*, 2004a; Korkolopoulou *et al*, 2005; Shetty *et al*, 2005). The MCM genes have also appeared as part of ‘poor’ prognostic signatures in breast cancer (van ‘t Veer *et al*, 2002; Sotiriou *et al*, 2003; Yu *et al*, 2004a), mantle cell lymphoma (Rosenwald *et al*, 2003) and medulloblastoma (Neben *et al*, 2004), whereas in cervical cancer, MCM protein expression appears promising as a predictor of response to radiation therapy (Mukherjee *et al*, 2001).

In conclusion, in the present study, we have investigated for the first time MCM-2 and MCM-5 expression in LMP tumours and ovarian adenocarcinomas in relation with clinicopathologic parameters, cell cycle modulators and patients’ survival. Both proteins associated significantly with high grade, advanced stage and residual disease as well as with Ki-67 proliferative index. MCM-2 LI was inversely related to CdK inhibitor p27^Kip1^, whereas both MCM-proteins correlated positively with p53 expression in carcinomas. MCM-2 and MCM-5, but not Ki-67, emerged as independent predictors of poor overall survival, implying that these molecules may be used to refine the prognostic information conveyed by standard predictors of outcome. On the basis of these findings, the tempting possibility of targeting replication proteins and their regulators as part of antitumour therapies that interfere with cancer cell proliferation certainly merits consideration.

## Figures and Tables

**Table 1:** Clinical data of patients in the study population with ovarian LMP tumours and adenocarcinomas

*Age (years)*	
Median (range)	51.6 (20–86)

*Histological type*	
LMP tumours	
Serous	29 (67.4%)
Mucinous	14 (32.6%)
Adenocarcinomas	
Serous	34 (40%)
Mucinous	10 (11.76%)
Endometrioid	16 (18.82%)
Clear cell	18 (21.17%)
Poorly differentiated	3 (3.5%)
Not specified	4 (4.7%)

*Histologic grade*	
1	13 (15.3%)
2	240 (47.1%)
3	32 (37.6%)

*FIGO stage*	
I	25 (29.5%)
II	3 (3.5%)
III	42 (49.4%)
IV	11 (12.9%)
Not specified	4 (4.7%)

*Primary residual tumour*	
No	39 (45.9%)
Yes	46 (54.1%)

*Adjuvant chemotherapy*	
Platinum-containing	64 (75.3%)
Non platinum-containing	21 (24.7%)

*Clinical outcome*	
Died of ovarian cancer	26 (30.6%)
Alive	53 (62.4%)
Not specified	6 (0.7%)

**Table 2:** Clinical data of patients in the validation group with ovarian adenocarcinomas

*Age (years)*	
Median (range)	58 (22–86)

*Histological type*	
Serous	35 (77.8%)
Mucinous	1 (2.2%)
Endometrioid	3 (6.7%)
Clear cell	3 (6.7%)
Poorly differentiated	1 (2.2%)
Not specified	2 (4.4%)

*Histologic grade*	
1	4 (9.3)
2	20 (46.5)
3	19 (44.2%)

*FIGO stage*	
I	1 (2.2%)
II	6 (13.3%)
III	29 (64.4%)
IV	9 (20.0%)

*Primary residual tumour*	
No	16 (35.6%)
Yes	28 (62.2%)
Not specified	1 (2.2%)

*Adjuvant chemotherapy*	
Platinum-containing	34 (75.6%)
Non platinum-containing	11 (24.4%)

*Clinical outcome*	
Died of ovarian cancer	29 (64.4%)
Alive	16 (35.6%)

**Table 3:** MCM-2 and MCM-5 expression in LMP tumours and ovarian adenocarcinomas

**Histology**	**MCM-2 LI (%) median (range)**	**MCM-5 LI (%) median (range)**
*LMP tumours, n=42*		
Serous	5.5 (1–25)	10 (2–20)
Mucinous	5 (1–15)	5 (1–20)

*Adenocarcinomas, n=72*		
Serous	20 (2–60)	22.5 (7–70)
Mucinous	20 (8–35)	24 (15–70)
Endometrioid	17.5 (8–82)	27.5 (8–80)
Clear cell	15 (2–70)	20 (5–50)
Poorly differentiated/Not specified	35 (20–50)	40 (10–75)

**Table 4:** MCM-2 and MCM-5 expression as related to clinicopathologic variables in ovarian carcinomas

**Variable**	**MCM-2 LI (%) median (range)**	**MCM-5 LI (%) median (range)**
*Histological grade*		
1	10 (2–50)	30 (3–70)
2	17.5 (5–45)	20 (5–60)
3	30 (5–82)	42.5 (5–80)

*Kruskal–Wallis test*	*P*=0.0002	*P*=0.0006

*Stage*		
I–II	15 (2–50)	20 (3–75)
III–IV	20 (5–82)	35 (5–80)

*Mann–Whitney U test*	*P*=0.0062	*P*=0.0018

*Residual tumor*		
No	15 (2–45)	20 (3–70)
Yes	27.5 (5–82)	35 (10–80)
*Mann–Whitney U test*	*P*=0.0030	*P*=0.0010

**Table 5:** Cox proportional hazard estimation of overall survival in ovarian adenocarcinomas

			**95% confidence limits for the hazard ratio**	
**Covariate**	***P***	**Hazard ratio**	**Lower**	**Upper**
*A1* a				
MCM-2	0.001	1.058	1.023	1.093
Stage (I/II *vs* III/IV)	0.045	11.16	1.06	117.52

*B1* b				
MCM-5	0.036	1.027	1.001	1.053

*A2* c				
MCM-2	<0.0001	1.116	1.049	1.187
p21^WAF1^	0.028	0.621	0.406	0.949

*B2* d				
MCM-5	0.022	1.043	1.006	1.082

a A1 model included the conventional clinicopathologic parameters and MCM-2LI.

b B1 model included the conventional clinicopathologic parameters and MCM-5LI.

c A2 model included the conventional clinicopathologic parameters, MCM-2LI, Ki-67LI and the cell cycle regulators.

d B2 model included the conventional clinicopathologic parameters, MCM-5LI, Ki-67LI and the cell cycle regulators.
